# Topology, landscapes, and biomolecular energy transport

**DOI:** 10.1038/s41467-019-12700-w

**Published:** 2019-10-11

**Authors:** Justin E. Elenewski, Kirill A. Velizhanin, Michael Zwolak

**Affiliations:** 1 Biophysics Group, Microsystems and Nanotechnology Division, Physical Measurement Laboratory, National Institute of Standards and Technology, Gaithersburg, MD 20899 USA; 2 Maryland Nanocenter, University of Maryland, College Park, MD 20742 USA; 3 Theoretical Division, Los Alamos National Laboratory, Los Alamos, NM 87545 USA

**Keywords:** Computational biophysics, Energy transfer, Molecular dynamics, Biological physics

## Abstract

While ubiquitous, energy redistribution remains a poorly understood facet of the nonequilibrium thermodynamics of biomolecules. At the molecular level, finite-size effects, pronounced nonlinearities, and ballistic processes produce behavior that diverges from the macroscale. Here, we show that transient thermal transport reflects macromolecular energy landscape architecture through the topological characteristics of molecular contacts and the nonlinear processes that mediate dynamics. While the former determines transport pathways via pairwise interactions, the latter reflects frustration within the landscape for local conformational rearrangements. Unlike transport through small-molecule systems, such as alkanes, nonlinearity dominates over coherent processes at even quite short time- and length-scales. Our exhaustive all-atom simulations and novel local-in-time and space analysis, applicable to both theory and experiment, permit dissection of energy migration in biomolecules. The approach demonstrates that vibrational energy transport can probe otherwise inaccessible aspects of macromolecular dynamics and interactions that underly biological function.

## Abstract (web summary)

Understanding vibrational energy transfer in macromolecules has been challenging to both theory and experiment. Here the authors use non-equilibrium molecular dynamics to reveal the relationship between heat transport in a model peptide, emergent nonlinearity, and the underlying free energy landscape.

## Introduction

Biological systems are characterized by a persistent non-equilibrium state, maintained by the open metabolic reactions that drive self–replication. Directed redistribution of energy is an intrinsic feature, serving to generate mechanical motion^1,2^, mediate allosteric communication^3–5^, and drive bioenergetic processes^6–8^. The physical scales of these processes can be surprising: Common enzymatic reactions liberate up to 2 eV of heat repeatedly over micro– to milli–second catalytic cycles^8^. This energy is redistributed throughout the surrounding protein scaffold within picoseconds and is either dissipated to mitigate thermally–induced stress, leveraged to induce mechanical motion, or employed to promote further catalytic activity. Irrespective of the endpoint, efficient and directed energy transport is critical to the function of these nanoscale machines.

At the macroscale, Fourier’s law, *J* = −*κ*∇*T* and its time–dependent version capture diffusive heat flow, given by the flux *J*, in response to a temperature gradient ∇*T*. Those two quantities are related by the thermal conductivity *κ* (or the diffusivity *D*), which can be anisotropic. This situation is more complicated at the nanoscale, where competing ballistic and diffusive transport pathways impede a universal description^9,10^. In this context, ballistic wavepackets propagate at the speed of sound in a given vibrational band, up the vibrational mean free path, even without the local thermal gradients required for diffusive transport.

Despite the ubiquity of energy redistribution and flow in biomolecular systems, experiments are difficult^6,11–15^. In a pioneering work, Botan et al.^12^ developed an approach to monitor real–time heat migration in a polypeptide of 2–aminoisobutyric acid (Aib). The approach employs a photoexcitable azobenzene tag as a heater and backbone carbonyl modes as local vibrational thermometers. The results are complex, suggesting a ‘dynamical transition’ temperature above which transport is enhanced^16–19^. Quantum and non-equilibrium molecular dynamics (NEMD) simulations support the presence of a transition in transport properties, and also suggest that a classical description is realistic^20–23^ (unlike for small molecules^24–28^). However, both the nature of the transition and mechanism of transport remain unclear, with theory giving conflicting accounts^12,16,20,21,29^.

In this work, we utilize molecular dynamics simulations and a new space- and time-local analysis method to explore energy propagation in a paradigmatic polypeptide. We find that Fourier behavior captures the bulk of transient energy flow, provided that one accounts for the fact that fluxes and diffusivities are temperature dependent. Departures from a simple realization of Fourier’s law happen at large temperature gradients, beyond about 15 K/residue, even though transport is still diffusive. The identification of these regimes is not possible through all-atom molecular dynamics alone^20–23,30–32^ or normal-mode analysis (even when treating anharmonicity as a correction)^33–39^. The former does not unravel the atomic-scale mechanisms of transport and the latter reflects dynamics only at potential energy minima^36^. Within this context, we further demonstrate how the graph–theoretic topology of molecular contacts can define directed pathways for molecular energy redistribution.

## Results

### Topology and energy propagation pathways

We initiate our investigations using a series of replica-exchange molecular dynamics (REMD) simulations, as the lack of symmetries, granularity, and high-dimensional free energy landscapes of biomolecules necessitate an exhaustive exploration of conformational space^40–42^.

**Fig. 1:**
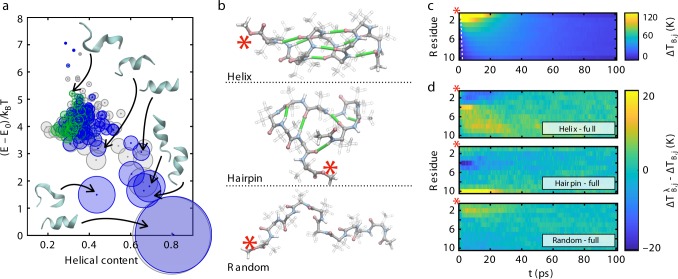
Free energy landscapes, topology, and energy transport. **a** Conformational clusters within the Aib_10_ free energy landscape at the solvent bath temperature *T*_B_ = 230.0 K. The size of a data point reflects the relative population of a *k*–means structural cluster at 2.6 nm root–mean–square deviation (RMSD) cutoff. States for a right–handed helix are colored from blue (more chiral) to green (less chiral), while those of a left–handed helix are uniformly gray. Helicity parameters and ensemble determinations follow ref. ^43^; **b** Major conformers in the Aib_10_ structural ensemble. The C–terminal heater residue is denoted by a red asterisk (*), and hydrogen bonds are colored green; **c** Thermal transport profile from NEMD simulations, characterized as a per–residue kinetic temperature elevation Δ*T*_B,*j*_(*t*) = 〈*T*_*j*_(*t*)〉 − *T*_B_ with respect to the solvent bath. The dashed, white line demarcates the ballistic front; **d** Differential heat transport between a full structural ensemble and those ($\Delta T_{{\mathrm{B}},j}^\lambda$) containing only *λ* = helical, hairpin, or unstructured populations. Upper and lower limits on the temperature elevation (e.g., on Δ*T*_B,*j*_) provide a cutoff for all values lying above or below the bound, respectively

Our simulation system is a ten-residue Aib helix (Aib_10_) solvated by chloroform, similar to experimental efforts^12,16–19^. We previously generated temperature-dependent free energy landscapes for Aib_10_ at high resolution with replica-exchange simulations^43^. From the resulting conformational ensemble, we extract 4000 conformers for each environmental (bath) temperature *T*_B_ according to a Boltzmann distribution. This includes structures from both left- and right-handed folding funnels, ensuring a uniform distribution of configurations (Fig. 1a, b). We initiate NEMD simulations in a manner that mimics photoexcitation, distributing ≈1.6 eV of energy between designated vibrational degrees of freedom in each conformer. This is achieved by thermostatting the C–terminal residue to a temperature *T*′ = *T*_B_ + Δ*T*, with Δ*T* = 670 K, while holding the remainder of the system at *T*_B_. The simultaneous heating of all vibrational degrees of freedom in the heater residue is well-founded, as it yields thermal transport profiles that are indistinguishable from mode-selective heating^12,20^. This excess energy then propagates freely within the microcanonical ensemble (i.e., without thermostatting).

The conformational ensemble of Aib_10_ comprises three general structural motifs (Fig. 1b) corresponding to (i) 3_10_–/*α*–helical conformers (≈45% of ensemble) with hydrogen bonding between residue *j* and residue *j* + 3 or *j* + 4, respectively; (ii) hairpin–like configurations, with hydrogen bonds between the first and last residues of Aib_10_ (≈15%); and (iii) unstructured or extended conformers that have no consistent hydrogen bonding (≈40%)^43^. We index these subensembles with *λ*. This partition is defined by the underlying free energy landscape, and is thus independent of our thermal transport simulations^43^.

In Fig. 1c, d, we present transport profiles for Aib_10_ versus the ensemble-averaged temperature elevation Δ*T*_B,*j*_(*t*) = 〈*T*_*j*_(*t*)〉 − *T*_B_ of the *j*^th^ residue, or $\Delta T_{{\mathrm{B}},j}^\lambda$ − Δ*T*_B,*j*_ for subensemble *λ*. The full-ensemble profile exhibits a weak thermal front that traverses the peptide within 2 ps, which is also apparent in the helical ensemble (Fig. 1d). This corresponds to backbone propagation at *v* = 1.7 nm ps^−1^, approaching ballistic transport velocities in biomolecular materials and alkyl chains^12,25–27,44^. While this channel is weak, additional ballistic pathways may exist at lower group velocities in different vibrational bands^44,45^, though these will inevitably be obscured by more prominent diffusive features. There is also rapid transport with both ballistic and diffusive characteristics across hydrogen-bonded regions, which can be seen in the helical and hairpin conformers (see discussion below).

**Fig. 2:**
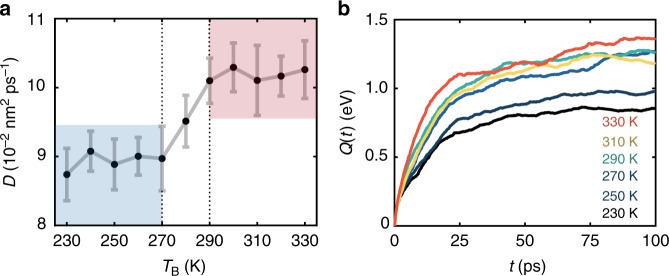
Benchmarks for thermal transport. **a** Heat diffusivity *D* along the major axis of helical Aib_10_ at increasing bath temperatures *T*_B_. Diffusivities are derived from the time *t*_max_ to reach the maximal temperature at each residue following a model *t*_max_ = *d*^2^/*D*, where *d* is the distance from the heater site. Colored regions denote low– (blue) and high–temperature (red) regimes (error bars are plus/minus one standard error). **b** Net heat *Q*(*t*) transferred from residue two to three versus simulation time and bath temperature, following the scheme of Eq. (1). Error bands for the maximal cumulative integration error, as well as net heat transfer between other residues, are in Supplementary Figs. 1–9

While a ballistic pathway exists, the majority of energy transport is nonetheless diffusive—yielding a broad profile that is sensitive to both temperature and molecular conformation. We separate diffusive and ballistic behavior by coarse-graining in time (into 100 fs bins), averaging away signatures of very fast dynamics, but retain spatial coarse-graining into individual amino acid residues. We will develop time-dependent quantitative methods to extract diffusivities, free energies, and other characteristics from temperature–based data. However, to facilitate comparison with prior theory and experiment, we initially calculate diffusivities via the time to reach the maximal temperature for each residue. Considering just the helical subensemble for fitting, the temperature-dependent thermal diffusivity *D*(*T*_B_) has distinct low- and high-temperature regimes (Fig. 2a), which are also reflected in the net heat transfer (Fig. 2b). This qualitative behavior agrees with experimental^12,17,19^ and theoretical^12,20,21^ efforts. These, though, report diffusivities of 0.02 and 0.1 nm^2^ ps^−1^, respectively. Theoretical *D*(*T*_B_) from this type of estimate consistently exceed experimental values for Aib_10_ but are comparable to bulk materials^27^ and other proteins^39^. Force-field parameterization likely contributes to this discrepancy in part. We will see, through an alternate analysis, that residual ballistic components also play a role. The crossover near 270 K is consistent with prior efforts, which ascribe this behavior to a glass-like dynamical transition^12,17,19,20^. We will return to this point.

Given this diverse ensemble, it is natural to ask how transport behaves in different conformers. This question was not addressed by prior computational efforts, as they remained below the timescale for structural interconversion in forming their ensemble, sampling only helical configurations and thus a fixed secondary connectivity^12,20^. Figure 1d shows the transport profile of the full Aib_10_ ensemble compared to ensembles that contain only helical motifs, hairpin motifs, or randomly oriented conformers without fixed secondary structure. On a residue-by-residue basis, helical conformers propagate heat more readily than the full ensemble. This is evidenced by less energy retention at the heater site for *t* ≤ 25 ps, commensurate with enhanced transfer to its hydrogen-bonded contacts at early times (mostly site 4 for the helix). The randomly oriented conformers transport heat less efficiently, underscored by enhanced energy localization at the first three residues for short times and, later, a rate of energy migration that lies slightly below the full ensemble. We expect a dominant backbone contribution in this case, as longer range contacts are sporadic. Hairpin configurations are intermediate, with enhanced transport to certain hydrogen bond contacts (site 10), in turn reducing the amount of heat transport through others (to the fourth site). It should be noted that, while hydrogen bonding can lead to more efficient heat transport for certain conformers, backbone channels always carry the majority of heat. Changes in energy migration are not due to local solvent heating, as the mean temperature of the first two solvation shells increases by at most 5 K over the entire simulation. While the overall cooling rate involves an interplay between heat diffusivity and surface area-dependent solvent coupling, these effects are minor for the systems considered herein (see the Supplementary Discussion).

These observations indicate that topologically nontrivial configurations yield efficient pathways for vibrational energy migration. The importance of secondary and tertiary contacts has been previously invoked when describing transport within a single conformer of HP36^32,39^. We extend this observation, demonstrating that representative heat transport characteristics can be obtained only when the conformational landscape is comprehensively sampled. This is particularly important for metrologies, where insufficient sampling can lead to erroneous diffusivities and the misidentification of transport pathways. Moreover, changing conditions (temperature, pH, presence of denaturants, etc.) can shift the conformational ensemble, particularly near structural transitions. This will be detected by the energy transport, including the capture of additional information about underlying interactions^46–48^.

### Heat fluxes and energy landscape topography

While molecular connectivity clearly determines transport pathways, NEMD simulations and existing analysis frameworks afford no immediate means to reconcile temperature-dependent features with microscopic processes and the underlying free energy landscape. To directly address this, we analyze the intermediate-timescale dynamics of NEMD trajectories—restricting to helical Aib_10_ conformers for both structural heterogeneity and consistency with prior work—using a master equation for the kinetic energy *E*_*j*_ of the *j*th residue in the peptide:

$$\dot E_j(t) = \mathop {\sum}\limits_i {[k_{ij}(t)E_i(t) - k_{ji}(t)E_j(t)]}$$

1 $$\hskip 14pt - \, k_{{\mathrm{s}},j}(t)[E_{{\mathrm{s}},j}(t) - E_j(t)].$$

In this case, *k*_*ij*_(*t*) is a rate constant for energy transfer from residue *i* to residue *j* and *k*_*ji*_(*t*) is a distinct rate for the reverse process (see Methods), *k*_s,*j*_(*t*) is the rate of heat transfer to the solvent bath, and *E*_s,*j*_(*t*) is the kinetic energy density of the solvent surrounding the *j*th residue (scaled to match the residue degrees of freedom). We diverge from earlier work by treating the *k*_*ij*_(*t*) as parameters that depend on both position and time—thereby implying a temperature dependence. This accommodation is key to our subsequent analysis. Given this arrangement, one can identify two distinct intra–peptide couplings: (i) direct transfer between nearest–neighbors in the peptide backbone (*k*_*j*,*j*+1_ and *k*_*j*,*j*−1_) and (ii) a long distance coupling between hydrogen bonding partners (*k*_*j*,*j*+3_, *k*_*j*,*j*+4_ for ideal 3_10_– and *α*–helices, respectively). With additional approximations, the system in Eq. (1) becomes well-posed and solvable at all times (see Methods). This diverges from existing master equation analyses, which assume rate constants that are time- and space-independent, and thus independent of the local temperatures and gradients^32^. These prior works nonetheless treat a broad network of nonlocal contacts, which combined with the analysis here would constitute a logical extension of our methods.

Our remaining discussion is driven by the pairwise heat fluxes *J*_*i*,*j*_(*t*_*n*_) = −*k*_*i*,*j*_(*t*_*n*_)[*E*_*i*_(*t*_*n*_) − (*f*_*j*_/*f*_*i*_)*E*_*j*_(*t*_*n*_)] and rate constants between coupled residues. Here *f*_*j*_ is the number of degrees of freedom for residue *j* and *t*_*n*_ indexes the time domain coarse-graining of the simulation trajectory into *n* ≤ *N* bins via block averaging. This approach is a finite difference decomposition of the diffusion equation $\dot E(x,t) = D{\mkern 1mu} \nabla ^2E(x,t)$ at the timescale Δ*t* = *t*_*n*+1_ − *t*_*n*_ and a length-scale Δ*x* defined by the distance between adjacent residues. The fluxes come from the finite difference decomposition of *J*(*x*, *t*) = −*D*∇*E*(*x*, *t*).

The rate constants *k*_*i*,*j*_(*t*_*n*_) = *D*(*t*_*n*_)/(Δ*x*)^2^, in particular, capture biomolecular heat diffusivity *D*(*t*_*n*_) while giving a metric for energy landscape features. We are interested in the distribution of barriers between low-lying conformational minima, specifically those connected by the energy-transmitting structural displacements that are associated with vibrational energy propagation. This latter property is reflected by the local, activated conformational changes underlying transport *k*_*i*,*j*_ = Ω_*i*,*j*_ exp[−Δ*G*_*i*,*j*_/*k*_B_*T*], where Δ*G*_*i*,*j*_ is the free energy barrier between heat-accepting microstates and (Ω_*i*,*j*_)^−1^ is an effective timescale for free diffusion, influenced by both the protein and its environmental coupling. While each pair of microstates is characterized by a distinct Δ*G*_*i*,*j*_, these values evolve during heat transport—commensurate with changes in the free energy landscape.

**Fig. 3:**
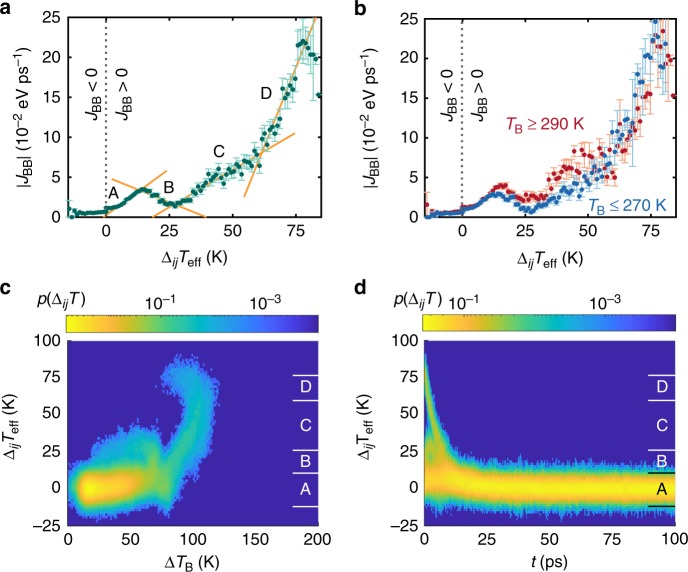
Flux and thermal gradient distributions. **a** Backbone flux distributions (*J*_BB_) for helical Aib_10_ conformers. Fluxes are parameterized by the effective temperature gradient Δ_*ij*_*T*_eff_ between adjacent residues, and a positive flux corresponds to flow away from the heater along the backbone. Transport regimes are labeled parallel to the text (A through D) and with orange lines for visibility. **b** The *J*_BB_ distribution may be partitioned into low–temperature (blue; 230–270 K) and high–temperature (red; 290–330 K) regimes. **c** Distribution of local temperature gradients Δ_*ij*_*T*_eff_ versus average elevation Δ*T*_B,*j*_(*t*) = 〈*T*_*j*_(*t*)〉 − *T*_B_ over the bath temperature and **d** versus simulation time *t* for the ensemble of MD trajectories. Labels (**a**–**d**) correspond to the regimes described in the text. Time series data from MD simulation is averaged with Δ*t* = 100 fs for fits to the master equation, Eq. (1), and the resulting fluxes are block averaged in 1.0 K bins. The error bars are plus/minus one standard error

We employ this kinetic approach with an intermediate timescale (Δ*t* = 100 fs), long enough to average over most coherent motion but short enough not to obscure the evolution of energy in time. The distribution of backbone fluxes *J*_BB_ is parameterized by an effective temperature gradient Δ_*ij*_*T*_eff_ = 2[*E*_*i*_ − (*f*_*j*_/*f*_*i*_)*E*_*j*_]/3*Nk*_B_ between residues *i* and *j*, where the flux is incident on a residue containing *N* atoms. While transport is explicitly quantified through *J*_BB_ for simplicity, the effect of hydrogen bonding is present when fitting the backbone flux distribution at hydrogen bonding sites. The results for *J*_BB_ are presented in Fig. 3a. A complimentary analysis for *J*_HB_ and a validation of fitting methods are presented in Supplementary Figs. 10–15.

### Region A

The forward flux *J*_BB_ has a linear region for small Δ_*ij*_*T*_eff_ (less than about 15 K), although it does not go to zero at Δ_*ij*_*T*_eff_ = 0. Purely diffusive transport will not afford a heat flux in the absence of a local temperature gradient. Thus, a finite *J*_BB_ at Δ_*ij*_*T*_eff_ = 0 is a signature of ballistic/coherent behavior. Supporting this interpretation, we find that the zero-gradient flux to decrease with increasing Δ*t* during coarse-graining, while only exhibiting small error bands at all scales (thus it is not due to short-timescale fluctuations). A linear fit to this regime gives an effective diffusivity of *D*_eff,A_ = 2.3 × 10^−2^ nm^2^ ps^−1^ (or conductivity *κ*_eff,A_ = 3.9 × 10^−3^ eV K^−1^ ps^−1^). Fitting for small Δ_*ij*_*T*_eff_, while ignoring the residual ballistic contribution right around Δ_*ij*_*T*_eff_ = 0, removes high rate constant artifacts. Encouragingly, the magnitude of the resulting diffusivity is consistent with experimental values^12,16^. Employing the time to reach the maximum temperature, as done in prior theoretical work (see discussion above), affords much higher diffusivities. This linear regime has the same slope regardless of whether the lattice is in the low- or high-temperature regime (Fig. 3b).

The lack of a dependence on temperature indicates that this regime of transport occurs in a lightly corrugated landscape—that is, with low-lying barriers separating the minima associated with thermal transport. In this case, the characteristic barrier scale is below 15 meV, and thus the mean energy at the lowest background temperature (*T*_B_ = 230 K) is above the landscape corrugation. Lower temperature observations are necessary to identify the precise scale, requiring an accurate treatment of quantum effects and different experimental protocols. Stated more succinctly, the equality of the low- and high-temperature diffusivity indicates that the characteristic time Ω^−1^ is the same and no free energy barrier exists at this level of landscape hierarchy.

### Region B

As Δ_*ij*_*T*_eff_ goes above 15 K, the flux decreases with the increasing temperature gradient. This suggests the appearance of a vibrational mismatch between adjacent residues due to nonlinearity. That is, adjacent residues separated by a sufficiently large temperature gradient will see different tiers of the energy landscape hierarchy and thus access different vibrational mode structures. As a consequence, the molecular conformation is pushed into an activated region of the free energy landscape where the energy barrier is larger than the available kinetic energy and increases with Δ_*ij*_*T*_eff_. Moreover, the average temperature elevation does not substantially change for Δ_*ij*_*T*_eff_ in region B where the flux dips (Fig. 3c). Thus, barrier crossing is not aided by energy remaining from the initial deposition. This is further supported by the separation of low- and high-temperature curves, indicating that transport increases with temperature—a signature of a free energy barrier. The characteristic barriers can be estimated from the ratio of high- and low-temperature fluxes (or rates), *J*_H_/*J*_L_ ≈ 1.2 ≈ exp(−Δ*F*/*k*_B_*T*_H_ + Δ*F*/*k*_B_*T*_L_), giving values of Δ*F* that span from 28 to 167 meV when we use the average temperature in each regime (i.e., *T*_L_ = 250 K and *T*_H_ = 310 K). These effective barriers are precisely the energy scale leading to conformational changes that restore efficacious vibrational coupling.

### Region C

As Δ_*ij*_*T*_eff_ increases beyond 30 K, there is a substantial increase in flux for both low- and high-temperature structures. In this case, a large Δ_*ij*_*T*_eff_ implies a larger average temperature elevation for a given residue pair (Fig. 3c), as large gradients are primarily found at early times (and near the heater site) when a substantial fraction of initially deposited energy is present (Fig. 3d). If we assume Ω remains the same, the temperature elevation Δ*T*_B,*j*_ is enough to once again put transport in a stable regime of the landscape at this level of hierarchy, with a typical barrier energy of 67 meV. This yields an approximately linear region for *J*_BB_ with a diffusivity *D*_eff,C_ = 1.9 × 10^−2^ nm^2^ ps^−1^ (*κ*_eff,C_ = 3.2 × 10^−3^ eV K^−1^ ps^−1^).

### Region D

Increasing Δ_*ij*_*T*_eff_ even further, beyond 50 K, leads to a transport region with a larger diffusivity *D*_eff,D_ = 8.0 × 10^−2^ nm^2^ ps^−1^ (*κ*_eff,D_ = 1.3 × 10^−2^ eV K^−1^ ps^−1^), corresponding to over-the-barrier diffusion. In this case, a new level of the energy landscape hierarchy becomes accessible, which would otherwise require strong activation at lower energies.

**Fig. 4:**
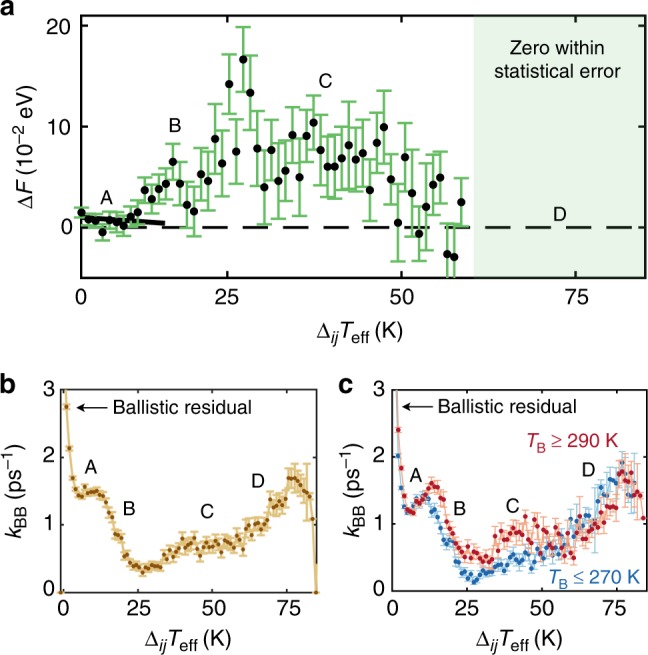
Transport barriers and kinetic parameters. **a** Effective free energy barriers Δ*F* corresponding to different regions of the *J*_BB_ flux profile. Region A has nearly no barrier, but as the gradient becomes large, a barrier starts to form and increases in region B. In C, this barrier decreases until in D it is zero to within statistical error (albeit, the uncertainty is large in this last region due to the limited number of samples for large temperature gradients, which occur only at short times). **b** Backbone rate distributions (*k*_BB_) for helical Aib_10_ conformers. Rate constants are parameterized by the temperature gradient Δ_*ij*_*T*_eff_ between adjacent residues and **c** partitioned into low-temperature (blue; 230–270 K) and high-temperature (red; 290–330 K) regimes. The error bars are plus/minus one standard error

Figure 4a shows the effective free-energy barriers in the different regimes, which are also reflected in the backbone rate constants (Fig. 4b, c). The *k*_BB_ initially decrease with Δ_*ij*_*T*_eff_ (from 0 to 4 K) due to a diminishing residual ballistic component when averaging at Δ*t* = 100 fs. Overestimation of this signature (e.g., through an improper coarse–graining scale), can lead to the discrepancies with experiment found in earlier theoretical analyses^12,20^. This is followed by a plateau in *k*_BB_ at about 1.5 ps^−1^ between 4 and 15 K, followed by a drop as the landscape is pushed into a new, barrier-dominated region. After this, though, the larger Δ_*ij*_*T*_eff_ correspond to a larger temperature elevation, bringing the events above the features in the energy landscape and raising *k*_*BB*_ further. Our methods extract the dependence on the local temperature gradients and, by spatiotemporal correlation, the temperature elevation. Beyond Δ_*ij*_*T*_eff_ = 77 K, the rate constants and fluxes decline sharply, reflecting very early dynamics where strong dynamical localization processes dominate. These barriers collectively define the energy scales, and thus the rate of diffusion in conformational space^49^, that is associated with the mechanical dynamics of heat propagation at different temperatures.

## Discussion

While our NEMD simulations support that a transition^12,17,19,20^ in diffusivity is present, they do not support that the transition happens solely due to the existence of energy barriers, as stated in refs. ^12,16,21^, or glassy dynamics (which is certainly the case but does not pinpoint the particular processes that occur here). Rather, the transition is due to the development of region C physics: Energy flow, which largely happens from 0 to 10 ps, is in the presence of large Δ_*ij*_*T*_eff_ (see initial time, high gradient line in Fig. 3d) on top of equilibrium fluctuations (Δ_*ij*_*T*_eff_ ≈ ±10 K). We interpret this to indicate that large gradients give a vibrational mismatch via nonlinear energy localization, introducing a barrier to energy transport. In this context, localization would then mediate the transition into a higher diffusivity regime—thereby suggesting an origin of the sharpness of the transition. The increase of the base temperature reduces the vibrational mismatch by pushing the dynamics onto a different level of the landscape hierarchy. Simultaneous Arrhenius activation and barrier reduction conspire to give a sharp transition. More extensive simulations are necessary to make this precise.

These findings demonstrate that energy transport gives quantitative information regarding the biomolecular free energy landscape, its nonlinearity, and overall connectivity. Going beyond what we present here, the experimental analogues of our simulations offer potential probes of structural transitions, where a temperature-dependent change in the transport profile is a manifestation of the graph-theoretic topology associated with molecular contacts and nonlinear interactions of the dominant conformer(s). In other words, thermal transport can be employed to devise ‘tomographies’ that provide a complementary mapping of biomolecular structure, conformational dynamics, and folding pathways. While dominated by local contacts and secondary structure within the simple Aib_10_ peptide, we expect higher aspects of fold (tertiary, quaternary) to define these dynamics in increasingly complex biomolecules. Furthermore, such probes might excel for highly fluctuating systems such as intrinsically disordered proteins (IDPs), where efficacious thermal transport may still persist (addressed in the Supplementary Discussion), or as a means to dissect local shifts in vibrational mode structure during molecular signaling or allostery. These dynamics have been impervious to other spectroscopies. Our approach provides the conceptual foundations and analysis tools that are directly applicable to experimental data, permitting the immediate interpretation of measurements that leverage local vibrational thermometry. In addition to the functional implications, the approach will also enable the development of a better understanding of what interactions look like at the atomic scale, and therefore better force-fields, and facilitate the design of nanodevices with directed, environmentally responsive heat transport mechanisms.

## Methods

### Molecular dynamics simulations

Our simulations consist of a modified Aib_10_ peptide (AcOHN-Aib_10_-COOCH_3_), embedded in a box of 922 chloroform molecules. Equilibration and ensemble generation are described in ref. ^43^. Prior to NEMD runs, structures are further equilibrated for 100 ps at each base (*T*_B_) temperature (NPT; time step *δt* = 1.0 fs) followed by a 50 ps run with shorter time step (NPT; *δt* = 0.1 fs). Using the final configurations, NEMD (NVT; *δt* = 0.1 fs) is initiated by heating the first residue of Aib_10_ to *T*_B_ + Δ*T* (Δ*T* = 670 K) for 1 ps, while holding the remaining atoms at *T*_B_. Thermostatting is then disabled and heat propagation monitored in the microcanonical ensemble. Similar thermostatting protocols have been established as surrogates for explicit photoexcitation^20,32^. NVT simulations employ a velocity Verlet integrator and modified Nosé–Hoover thermostat (damping = 100 fs), while NPT runs add a Martyna–Tobias–Klein barostat (damping = 1000 fs, eight member chain)^50–52^. Isotropic cell fluctuations are allowed for NPT runs and initial velocities are assigned according to a Gaussian distribution. Simulations employ CHARMM36 force-field parameters^53,54^, CHARMM pair potentials (without CMAP parameters, as rationalized in ref. ^43^), transferrable parameters for CHCl_3_^55^, PPPM electrostatics (force cutoff 6.95 × 10^−3^ pN; pair coupling rescaled at 1.0 nm, terminated at 1.35 nm) and the LAMMPS codebase^56^. We have adopted a thermostat timescale that is faster than backbone amide relaxation and azobenzene isomerization in order to preserve transport-relevant dynamics. While a slight overpopulation of long–range modes remains possible, it would only serve to underestimate the impact of nonlinear localization while overestimating ballistic signatures—thus leaving our conclusions unaffected.

### Kinetic fitting

While physically descriptive, the master equation, Eq. (1), is underdetermined when fitting the simulated transport profiles *E*_*j*_(*t*) = 3/2*N*_*j*_*k*_B_〈*T*(*t*)〉 for the *N*_*j*_ atoms of the *j*th residue. As a simplifying approximation, we relate forward and reverse rate constants *k*_*ij*_ = (*f*_*i*_/*f*_*j*_)*k*_*ji*_ through the degrees of freedom of each residue *f*_*j*_, as required for detailed balance to hold at equilibrium. We also restrict analysis to structurally homogeneous (helical) conformers, where the rate constants for hydrogen bond energy transfer *k*_*j*,*j*+3_ ≈ *k*_*j*,*j*+4_ ≈ *k*_HB_ and solvent coupling *k*_s,*j*_ ≈ *R*_*j*_*k*_s_ can be approximated as uniform (up to a fixed geometric factor *R*_*j*_ for the surface area of terminal residues). Under these conditions, we may fit the time dependence of the solvent *k*_s_ → *k*_s_(*t*) and peptide rate constants, *k*_*ij*_ → *k*_*ij*_(*t*) and *k*_HB_ → *k*_HB_(*t*), to account for the local temperature (which changes in time). This is in contrast to prior efforts that assume a uniform and time–independent backbone rate constant *k*_*j*,*j*+1_ = *k*_BB_^32^.

Rate constants **k**_*j*_ = (*k*_1,2_, …, *k*_*N*−1,*N*_, *k*_H_) at the *n*th simulation time step are estimated for the linear system of Eq. (1) though a constrained optimization

2 $${\mathbf{k}}(t_n) = \min _{{\mathbf{k}} \ge 0}\frac{1}{2}||{\mathbf{G}}(t_n)\cdot {\mathbf{k}} - {\mathbf{d}}(t_n)||^2$$

where **d**_*j*_(*t*_*n*_) = [*E*_*j*_(*t*_*n*_) − *E*_*j*_(*t*_*n*−1_)] + *k*_s_(*t*_*n*_)[*E*_*j*_(*t*_*n*_) − *E*_s_] captures energy redistribution among residues of the peptide. The matrix **G**(*t*) is similarly defined so that **G**_*i*,*j*_(*t*) = −**G**_*i*+1,*j*_(*t*) = −[*E*_*i*_(*t*) − *E*_*j*_(*t*)] accommodates backbone energy transport and ${\mathbf{G}}_{i,N}(t) = \mathop {\sum}\nolimits_\ell {[E_i - E_\ell ]}$ describes its hydrogen bonding counterpart to the *i*th residue. The solvent coupling rate $k_{\mathrm{s}}(t_n) = \mathop {\sum}\nolimits_j {[E_j(t_n) - E_j(t_{n - 1})]/[E_j(t_n) - E_{\mathrm{s}}(t_n)]}$ is then given by the energy exchanged between the peptide and the solvent at each time step (the solvent bath energy *E*_s_(*t*_*n*_) = 3*N*_*j*_*k*_B_*T*_B_/2 is treated a constant).

## Supplementary information

Supplementary Information

## Data Availability

The authors declare that all data supporting the findings in this manuscript are available within the paper and its supplementary information.
