# Supplementary material for: Topology, landscapes, and biomolecular energy transport
Source: Nat Commun. 2019 Oct 11;10:4662. doi: 10.1038/s41467-019-12700-w (PMC6789131; doi:10.1038/s41467-019-12700-w)
Supplement: Supplementary file 1 — Supplementary Information [file 41467_2019_12700_MOESM1_ESM.pdf]

Supplementary Information for
“Topology, Landscapes, and Biomolecular Energy Transport”

Elenewski, *et al.*

I. SUPPLEMENTARY FIGURES

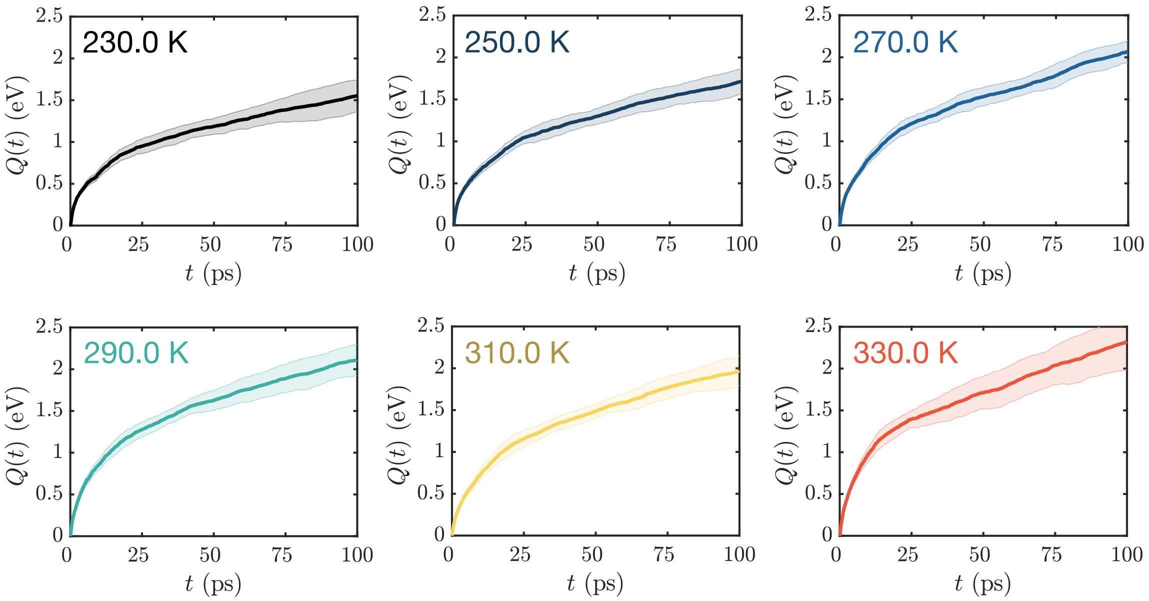

Supplementary Figure 1. Cumulative Heat Transfer Between Residues 1 and 2. Energy transfer is quantified by integrating the heat flux $[J_{\mathrm{BB}}]_{i,j}$ between the $i = 1$ and $j = 2$ residues up to time t in the MD simulation trajectory: $Q(t) = \int_0^t J_{1,2}(t')\, dt'$. Fluxes are obtained using the master equation analysis described in the parent manuscript, with time series data coarse grained at $\Delta t = 100$ fs prior to analysis. Transfers exceed the 1.6 eV of heat added to the first residue due to residual error from short timescale transients. This behavior may be mitigated by coarse graining the trajectory over larger temporal windows, at the cost of weaker statistics. The bath temperature for each trace (ΔT_{B}) is indicated in the upper left hand side of the plot. The error bands are plus/minus one standard error.

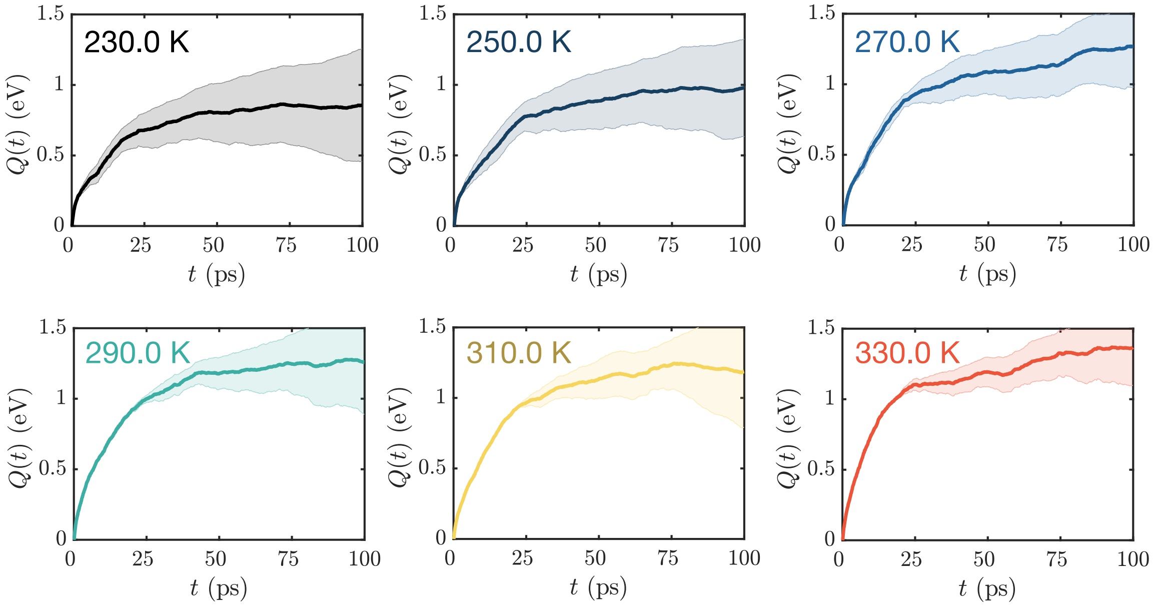

Supplementary Figure 2. Cumulative Heat Transfer Between Residues 2 and 3. Energy transfer is quantified by integrating the heat flux $[J_{\mathrm{BB}}]_{i,j}$ between the $i = 2$ and $j = 3$ residues up to time t in the MD simulation trajectory: $Q(t) = \int_0^t J_{2,3}(t')\,dt'$. Fluxes are obtained using the master equation analysis described in the parent manuscript, with time series data coarse grained at $\Delta t = 100$ fs prior to analysis. The bath temperature for each trace (ΔT_{B}) is indicated in the upper left hand side of the plot. The error bands are plus/minus one standard error.

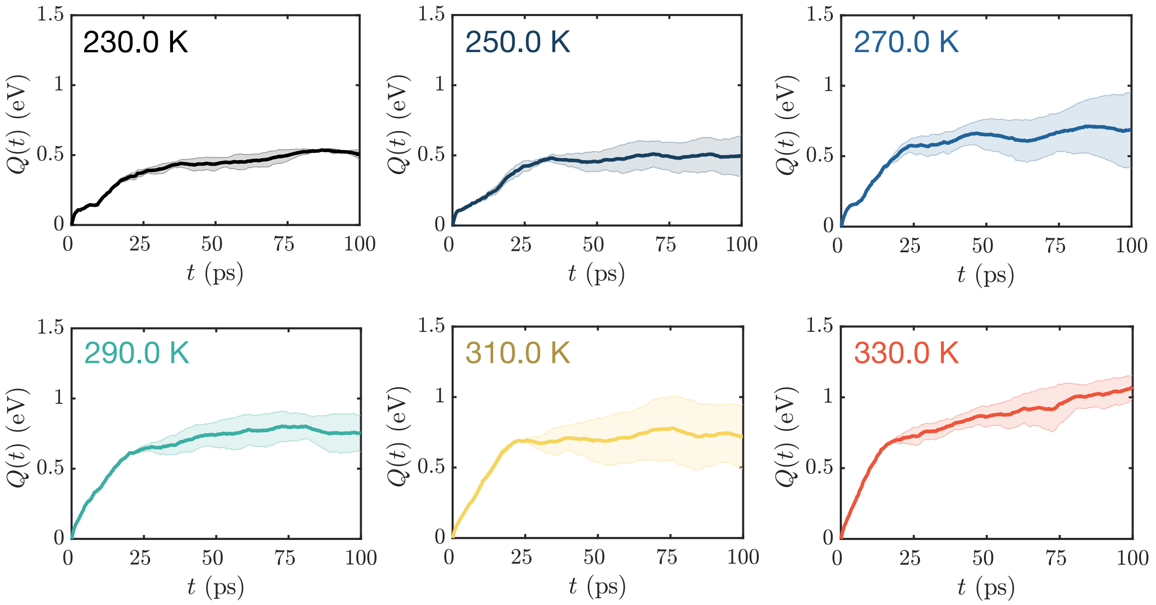

Supplementary Figure 3. Cumulative Heat Transfer Between Residues 3 and 4. Energy transfer is quantified by integrating the heat flux $[J_{\mathrm{BB}}]_{i,j}$ between the $i = 3$ and $j = 4$ residues up to time t in the MD simulation trajectory: $Q(t) = \int_0^t J_{3,4}(t')\,dt'$. Fluxes are obtained using the master equation analysis described in the parent manuscript, with time series data coarse grained at $\Delta t = 100$ fs prior to analysis. The bath temperature for each trace (ΔT_{B}) is indicated in the upper left hand side of the plot. The error bands are plus/minus one standard error.

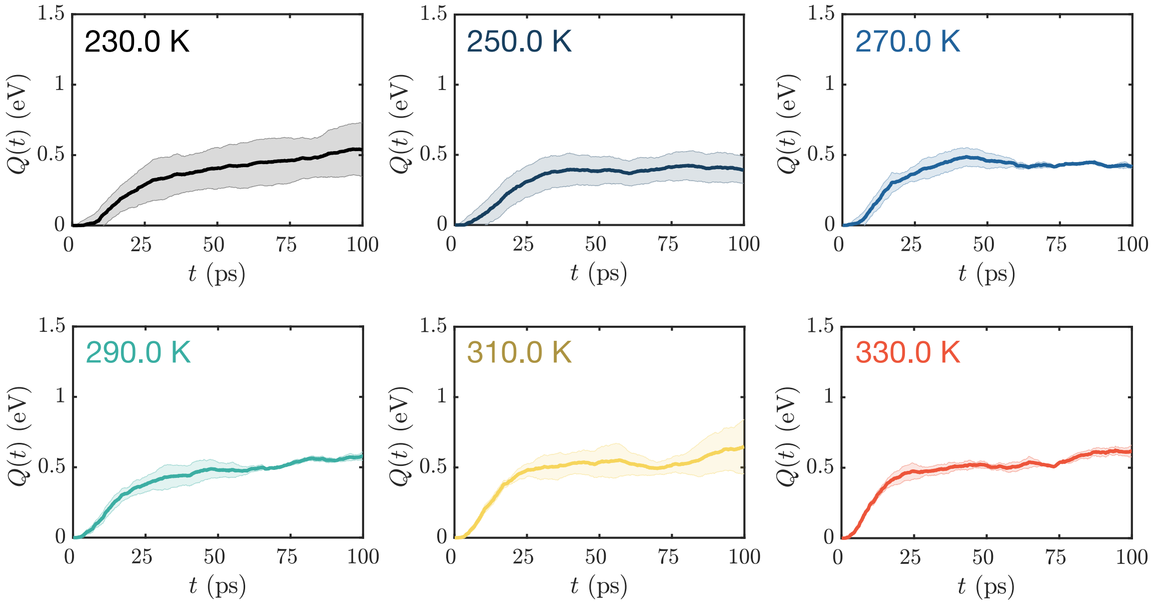

Supplementary Figure 4. Cumulative Heat Transfer Between Residues 4 and 5. Energy transfer is quantified by integrating the heat flux $[J_{\mathrm{BB}}]_{i,j}$ between the $i = 4$ and $j = 5$ residues up to time t in the MD simulation trajectory: $Q(t) = \int_0^t J_{4,5}(t')\,dt'$. Fluxes are obtained using the master equation analysis described in the parent manuscript, with time series data coarse grained at $\Delta t = 100$ fs prior to analysis. The bath temperature for each trace (ΔT_{B}) is indicated in the upper left hand side of the plot. The error bands are plus/minus one standard error.

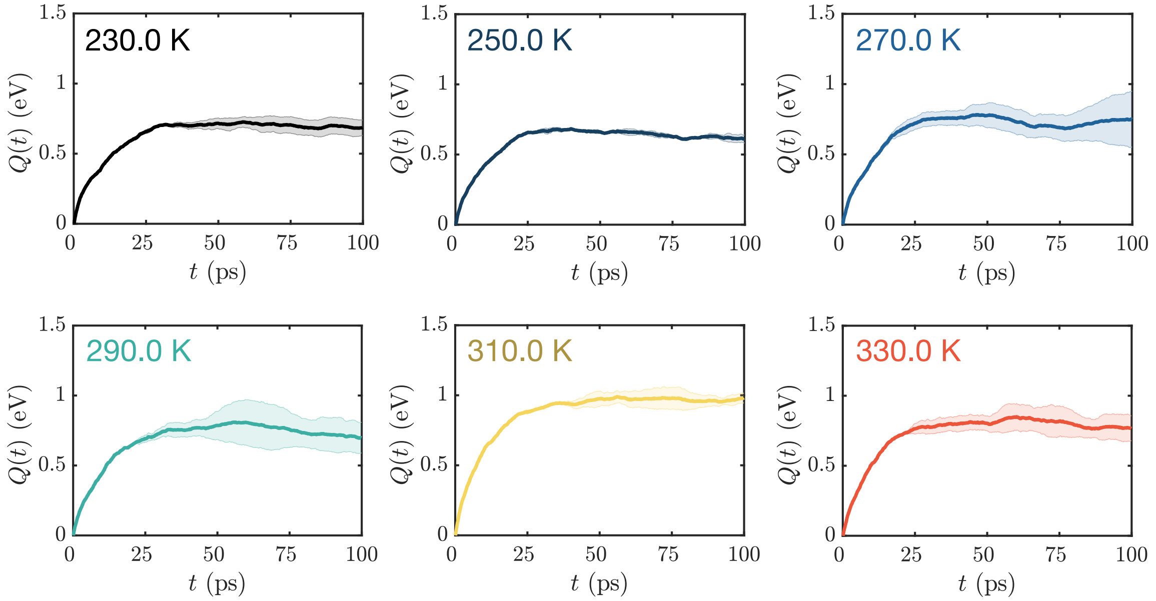

Supplementary Figure 5. Cumulative Heat Transfer Between Residues 5 and 6. Energy transfer is quantified by integrating the heat flux $[J_{\mathrm{BB}}]_{i,j}$ between the $i = 5$ and $j = 6$ residues up to time t in the MD simulation trajectory: $Q(t) = \int_0^t J_{5,6}(t')\,dt'$. Fluxes are obtained using the master equation analysis described in the parent manuscript, with time series data coarse grained at $\Delta t = 100$ fs prior to analysis. The bath temperature for each trace (ΔT_{B}) is indicated in the upper left hand side of the plot. The error bands are plus/minus one standard error.

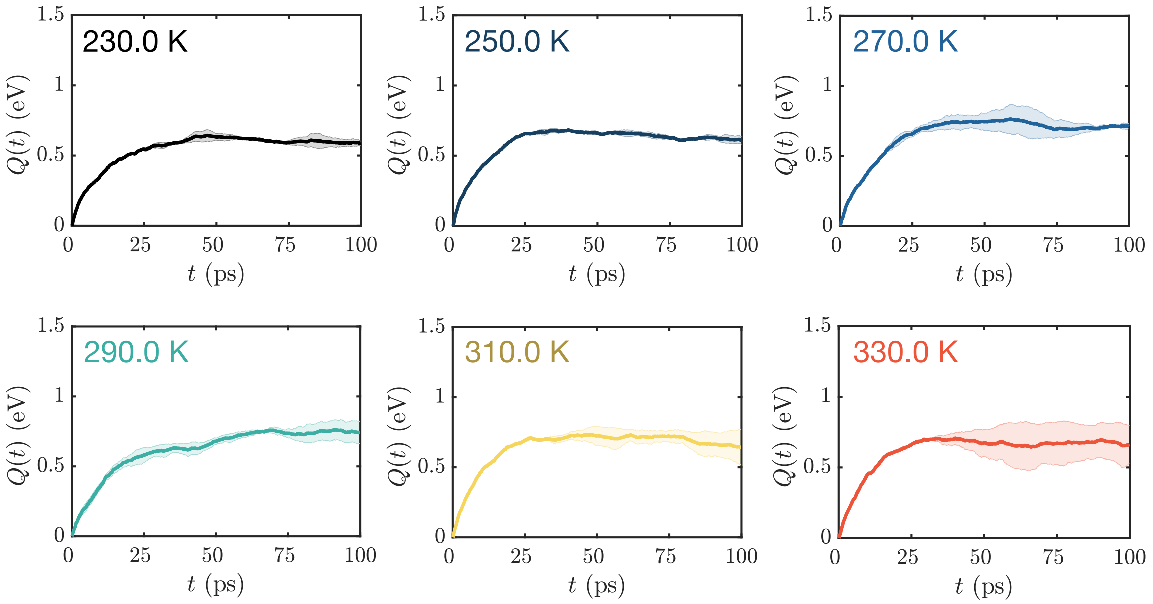

Supplementary Figure 6. Cumulative Heat Transfer Between Residues 6 and 7. Energy transfer is quantified by integrating the heat flux $[J_{\mathrm{BB}}]_{i,j}$ between the $i = 6$ and $j = 7$ residues up to time t in the MD simulation trajectory: $Q(t) = \int_0^t J_{6,7}(t')\,dt'$. Fluxes are obtained using the master equation analysis described in the parent manuscript, with time series data coarse grained at $\Delta t = 100$ fs prior to analysis. The bath temperature for each trace (ΔT_{B}) is indicated in the upper left hand side of the plot. The error bands are plus/minus one standard error.

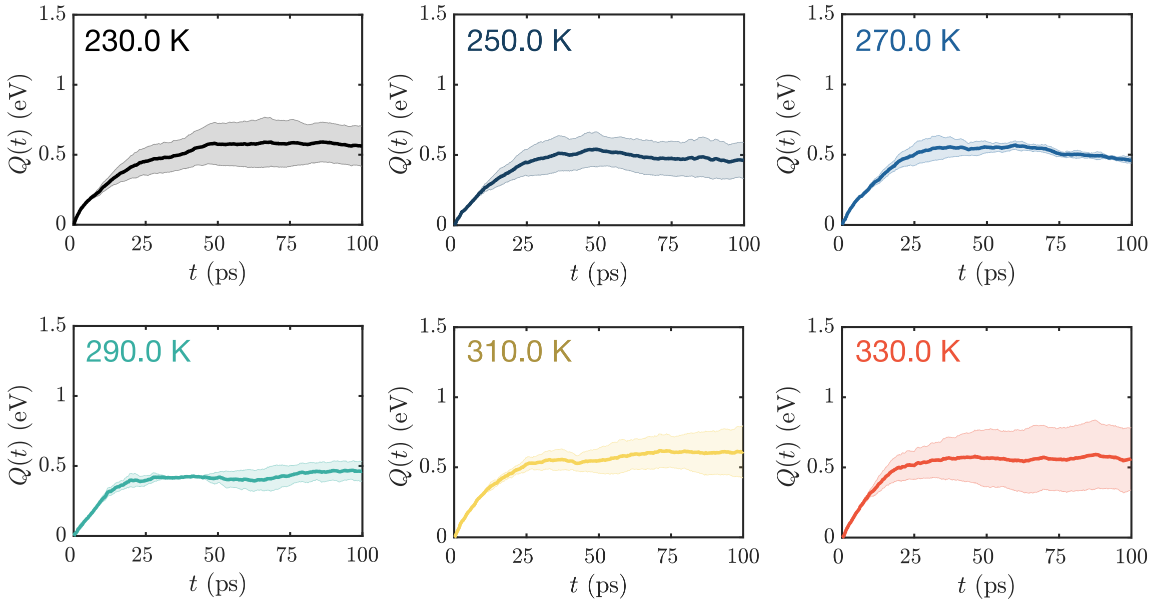

Supplementary Figure 7. Cumulative Heat Transfer Between Residues 7 and 8. Energy transfer is quantified by integrating the heat flux $[J_{\mathrm{BB}}]_{i,j}$ between the $i = 7$ and $j = 8$ residues up to time t in the MD simulation trajectory: $Q(t) = \int_0^t J_{7,8}(t')\,dt'$. Fluxes are obtained using the master equation analysis described in the parent manuscript, with time series data coarse grained at $\Delta t = 100$ fs prior to analysis. The bath temperature for each trace (ΔT_{B}) is indicated in the upper left hand side of the plot. The error bands are plus/minus one standard error.

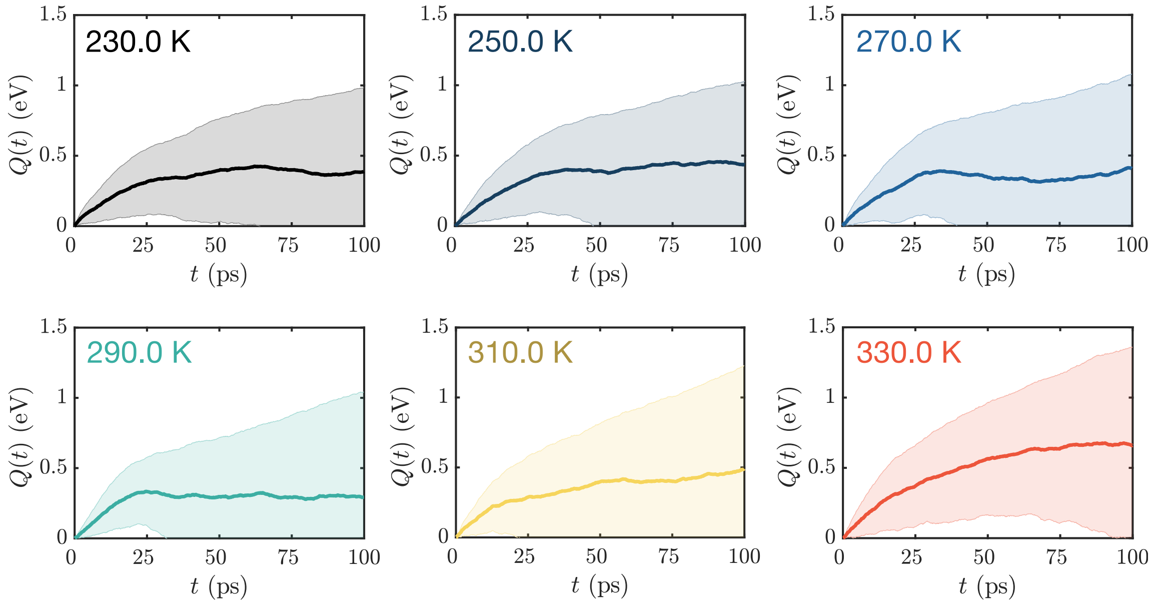

Supplementary Figure 8. Cumulative Heat Transfer Between Residues 8 and 9. Energy transfer is quantified by integrating the heat flux $[J_{\mathrm{BB}}]_{i,j}$ between the $i = 8$ and $j = 9$ residues up to time t in the MD simulation trajectory: $Q(t) = \int_0^t J_{8,9}(t')\,dt'$. Fluxes are obtained using the master equation analysis described in the parent manuscript, with time series data coarse grained at $\Delta t = 100$ fs prior to analysis. The bath temperature for each trace (ΔT_{B}) is indicated in the upper left hand side of the plot. The error bands are plus/minus one standard error.

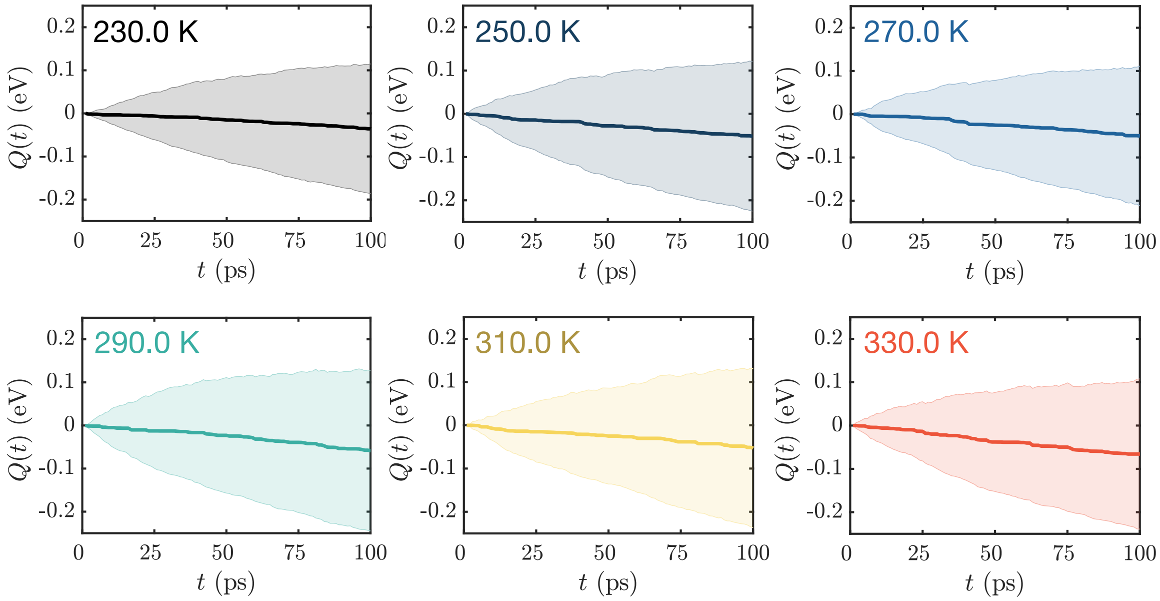

Supplementary Figure 9. Cumulative Heat Transfer Between Residues 9 and 10. Energy transfer is quantified by integrating the heat flux $[J_{\mathrm{BB}}]_{i,j}$ between the $i = 9$ and $j = 10$ residues up to time t in the MD simulation trajectory: $Q(t) = \int_0^t J_{9,10}(t')\,dt'$. Fluxes are obtained using the master equation analysis described in the parent manuscript, with time series data coarse grained at $\Delta t = 100$ fs prior to analysis. There is a small negative $Q(t)$. This is within the error, but may be due to the high flexibility of residue 10 leading to intermittent contact with the remainder of the peptide even with in the helical structure. This could give a thermal transport pathway from residue 10 to 9, or some residual effect from the solvent. The error bands are plus/minus one standard error.

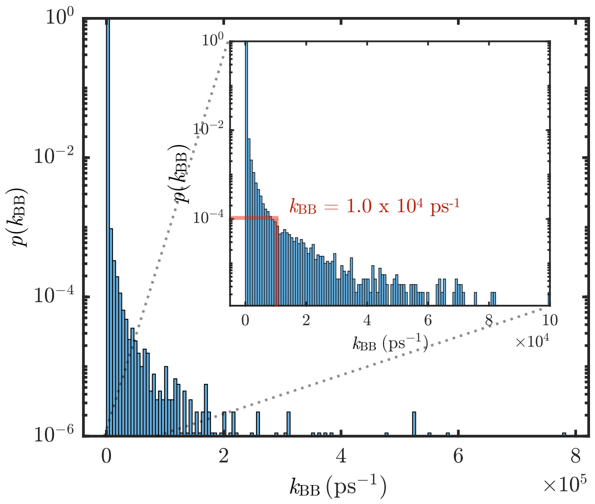

Supplementary Figure 10. Rate constant distribution. Probability density distribution $p(k_{\mathrm{BB}})$ for backbone rate constants k_{BB}, as obtained using the master equation–based fitting method (Equation 1 of the parent manuscript). Physically extremal values, defined as rates that exceed 6.2×10^3 ps^{-1}, have a statistical weight of less than 1.0×10^{-4}, attesting to the robustness of the fitting algorithm. The distribution is presented as an aggregate for helical conformers at all temperatures, with simulation time series data coarse grained over $\Delta t = 100$ fs intervals prior to analysis.

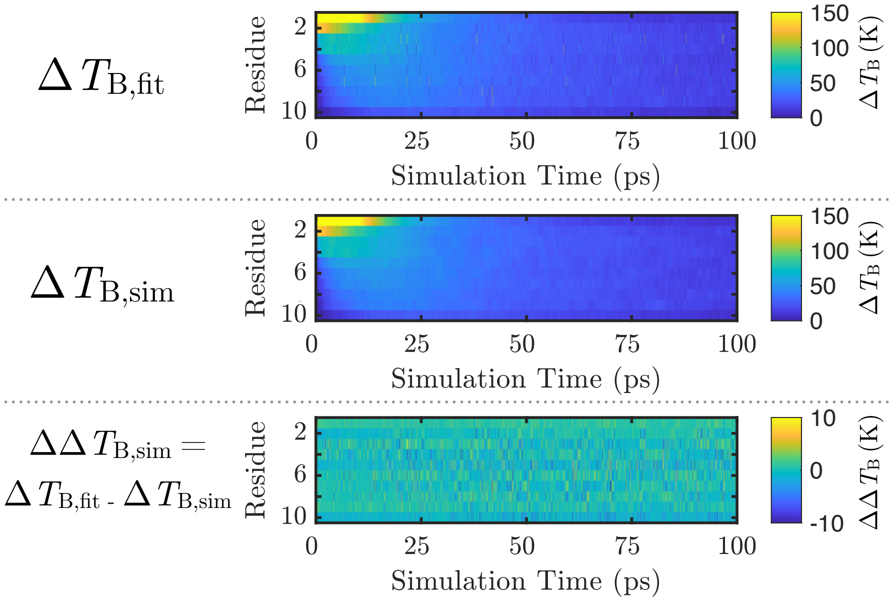

Supplementary Figure 11. Reconstructed thermal transport profiles. To demonstrate robustness of our master equation reconstruction (Equation 1 of the manuscript), molecular dynamics simulation profiles $\Delta T_{\mathrm{B,sim}}$ are propagated forward by one analysis timestep (Δt = 100 fs) using the backbone rate constants k_{BB} fit at that step. In doing so, we generate a temperature elevation profile $\Delta T_{\mathrm{B,fit}}$ for fit data. The deviation between fit and simulation data $\Delta\Delta T_{\mathrm{B}} = \Delta T_{\mathrm{B,fit}} - \Delta T_{\mathrm{B,sim}}$ affords a metric for quality of reconstruction, exhibiting variations that are generally below $\pm$10.0 K (Supplementary Figure 12). Data are presented for simulations at T_{B} = 230.0 K, with MD simulation data coarse grained over Δt = 100 fs intervals prior to analysis.

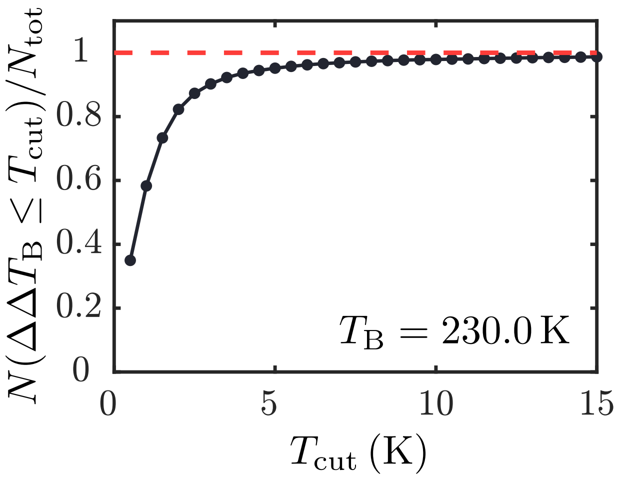

Supplementary Figure 12. Percentage of trajectory frame deviations by temperature cut-off. The variation between fit rate profiles and raw MD simulation (Supplementary Figure 11) may be further quantified through the percentage of trajectory propagations N/N_{tot} with a temperature deviation $\Delta\Delta T_{\mathrm{B}} = \Delta T_{\mathrm{B,fit}} - \Delta T_{\mathrm{B,sim}}$ lying at or below a given cutoff $\Delta\Delta T_{\mathrm{B}} \leq T_{\mathrm{cut}}$ (here N_{tot} is the total number of propagations). In this case, 95.2 % of propagations exhibit a deviation of less than 5.0 K over 100 fs, while 98.0 % show a deviation of less than 10.0 K over the same time interval. At deviations up to $T_{\mathrm{cut}} = 15.0$ K, we find that 99.0 % of propagations will lie below the cutoff. While not employed here, these values can be used to filter erroneous fits during analysis. In this case, $T_{\mathrm{cut}} = 10.0$ K would account for pathologies due to numerical instability while retaining robust counting statistics. Data are presented for simulations at $T_{\mathrm{B}} = 230.0$, with MD simulation data coarse grained over $\Delta t = 100$ fs intervals prior to analysis.

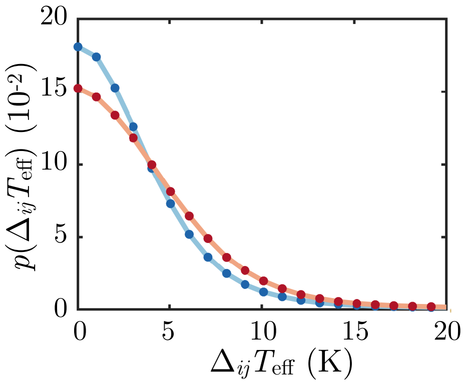

Supplementary Figure 13. Distribution of backbone temperature gradients. The probability density distribution $p(\Delta_{ij}T_{\rm eff})$ for the effective temperature gradient $\Delta_{ij}T_{\rm eff}$ between adjacent residues (taken as positive when $i > j$) is calculated directly from simulation data. Data are partitioned into low–temperature (blue; 230 K to 270 K) and high–temperature (red; 290 K to 330 K) regimes. Distributions are calculated using trajectory frames that have been coarse grained over $\Delta t = 100$ fs intervals, and are themselves averaged into 1.0 K bins.

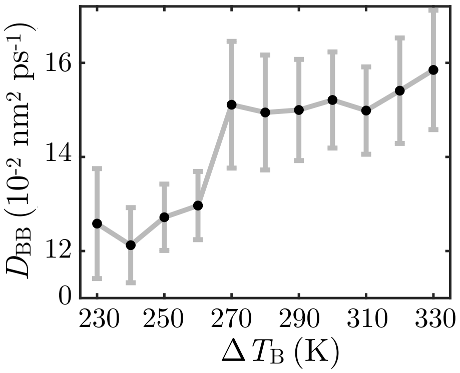

Supplementary Figure 14. Heat diffusivities calculated from backbone rate constants. The effective backbone heat diffusivity $D_{\rm BB} = k_{\rm BB}\,(\Delta x)^2$ may be estimated using the rate constants $k_{\rm BB}$ from the master equation analysis. Diffusivities are calculated as $D_{\rm BB}(T_{\rm B}) = \sum_{\ell=1}^{N} k_{{\rm BB},\ell}\, p_\ell\, (\overline{\Delta x})^2$ at each bath temperature $T_{\rm B}$, where the summation is over all N bins of the thermal gradient $\Delta_{ij}T_{\rm eff}$ distribution, p_ℓ is the weight assigned to each bin (Supplementary Figure 13), and $\overline{\Delta x}$ is the mean residue separation. The resulting diffusivities exhibit scaling that mimics the full simulation data (Fig. 2a in the manuscript), albeit with a slightly larger magnitude. While deviations exist (particularly for $T_{\rm B}$ = 240 K), these likely reflect the simplified interactions accommodated by our model, alongside limitations due to sampling. The overall similarity suggests that most critical processes are captured by the master equation approach, supporting the scope of our interpretation. The error bars are plus/minus one standard error.

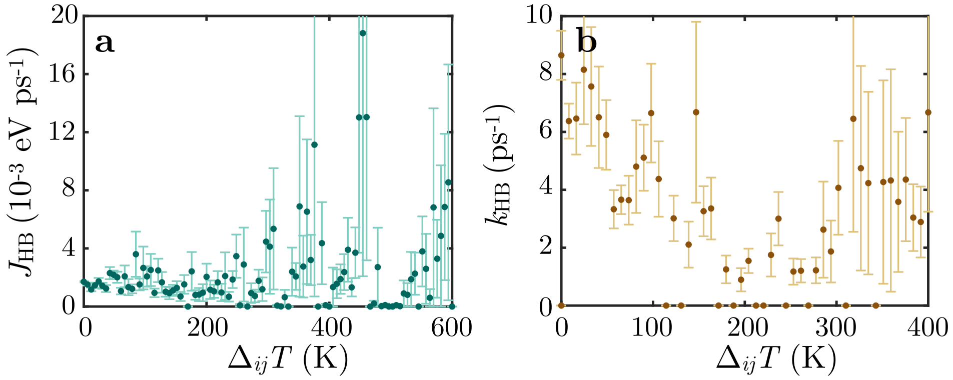

Supplementary Figure 15. Hydrogen Bond Rates and Fluxes. (a) Hydrogen bond flux distributions (J_{HB}) for helical Aib$_{10}$ conformers. Fluxes are parameterized by the effective temperature gradient $\Delta_{ij}T_{\mathrm{eff}}$ between adjacent residues, taken as positive when $i > j$. (b) Rate constants (k_{HB}) corresponding to the hydrogen bond flux distribution. Rates and fluxes are obtained by fitting a kinetic master equation (Equation 1 from the parent manuscript) to MD simulation data that has been coarse grained over $\Delta t =$ 100 fs intervals. The resulting fluxes and rates are block averaged into 1.0 K bins. The error bars are plus/minus one standard error.

II. SUPPLEMENTARY DISCUSSION

As a general rule, different folds of the Aib_{10} peptide (or other biomolecules) have a conformationally–dependent solvent–accessible surface area (SASA). Since thermal conduction between the peptide and the solvent bath occurs at this interface, it is expected that the overall thermalization profile will also depend on molecular conformation. To quantify this, we exploit the largely diffusive nature of heat conduction in Aib_{10} to construct a simple model for thermal relaxation. In this case, we assume two compartments — consisting of the peptide and the solvent — which undergo strictly conductive heat transfer according to Fourier's law (i.e., no convective contribution). Assuming that the solvent bath is much larger than the peptide, with minimal local solvent heating, we can write the total kinetic energy content $E(t)$ of Aib_{10} in the time–dependent form

$$E(t) = E_{\mathrm{B}} + [E(0) - E_{\mathrm{B}}]e^{-k_{\mathrm{c}}t}, \tag{1}$$

where E_{B} is the net kinetic energy content of Aib_{10} when in thermal equilibrium with the bath, $E(0)$ is the net kinetic energy content of peptide immediately following heating, and k_{c} is a characteristic time constant for heat transfer to the solvent. Using this expression, we employ three NEMD ensembles, containing 750 simulations each (at $T_{\mathrm{B}} = 230.0$ K), for helix, hairpin, and completely extended Aib_{10} conformers. The fits resulting from this protocol are depicted in Supplementary Figure 16 and summarized in Supplementary Table 1.

Our simple cooling model is generally robust when applied to thermal relaxation in Aib_{10}. Modest deviations between the resulting fits and simulation data are observed at early times ($t \leq 2.5$ ps) and high temperatures, where ballistic processes likely shunt heat to the solvent more rapidly than allowed by a diffusive mechanism. Outside of this region, the thermal transport dynamics are relatively similar for the helix, hairpin, and extended coil, with the most prominent (relative) conformational variation observed for the cooling rate constant k_{c}. In our simulations, the extended coil dissipates heat most rapidly to solvent, followed by the structured α–helix fold and comparatively globular hairpin conformations. While statistically significant, this effect is also small — largely due to the fact that Aib_{10} dynamics are surface–dominated in any conformer due to finite–size effects.

The overall scaling trend for k_{c} is mirrored when comparing the mean SASA between conformers (Supplementary Table 1). Quantitative differences nonetheless exist between SASA and k_{c} ratios [k_{c}(coil)/k_{c}(hairpin) = 1.10, while we find SASA(coil)/SASA(hairpin) = 1.26], with the surface

area contribution underestimated in the rate constants. While a variety of factors may collude in this effect, the conformer–dependent heat transport rates within the peptide likely make the largest contribution. That is, the helical and hairpin conformations possess high thermal diffusivities and auxiliary conduction pathways (due to molecular topology), which facilitate the transfer of heat to away from the heater and throughout the peptide (Supplementary Figure 17). Since the local cooling rate $\dot{E}_j(t)$ at the j–th residue is proportional to the local temperature gradient between the peptide and the solvent $\dot{E}_j(t) \propto T_j(t) - T_{\mathrm{s},j}(t)$, this accelerates solvent relaxation. The peptide thus acts as a radiator, relinquishing heat to the bath while circumventing local solvent heating — and thus partially mitigating the insulating effect of molecular conformation. We expect the contribution of this effect to be less in larger biomolecules — in a manner that depends on the surface–to–volume ratio — where redistribution pathways may also lead deeper into the molecular 'bulk' and thus away from the solvent interface.

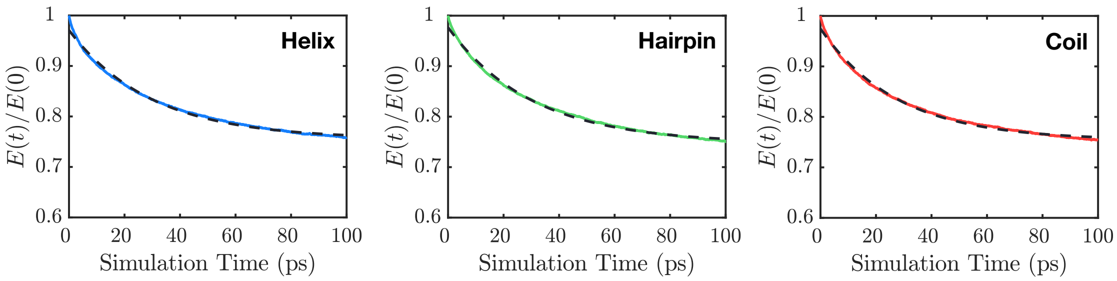

Supplementary Figure 16. Conformation–dependent cooling. Application of the the cooling model $E(t) = E_{\mathrm{B}} + [E(0) - E_{\mathrm{B}}]e^{-k_{\mathrm{c}}t}$ to NEMD simulations of distinct Aib_{10} conformers at $T_{\mathrm{B}} = 230.0$ K. Non-linear least–squares fitting was performed to the mean kinetic energy profile of each NEMD ensemble (each containing 750 distinct conformations) with block averaging into 10 fs bins prior to analysis. Error bands corresponding to the block standard error are displayed alongside simulation data (colored lines; bands are on the order of the line width), while the fits are depicted as a black dashed line. Data are measured relative to the peak net kinetic energy of Aib_{10}, with simulation methods identical to the parent manuscript.

Conformer	$E_B/E(0)$	$[E(0) - E_B]/E(0)$	k_c (ps^{-1})	SASA (nm^2)
Helix	0.7555 ± 0.0007	0.2153 ± 0.0009	0.0338 ± 0.0004	9.8 ± 0.14
Hairpin	0.7474 ± 0.0007	0.2293 ± 0.0008	0.0329 ± 0.0004	9.8 ± 0.16
Coil	0.7539 ± 0.0007	0.2209 ± 0.0009	0.0362 ± 0.0004	12.3 ± 0.15

Supplementary Table 1. Cooling fit parameters. Parameters for the cooling model $E(t) = E_B + [E(0) - E_B]e^{-k_c t}$, measured with respect to the peak kinetic energy content $E(0)$ of Aib_{10} immediately following excitation. Data is provided for 750–member ensembles of helix, hairpin, or extended coil conformers at $T_B = 230.0$ K, with the mean transport profile coarse–grained into 10 fs bins prior to analysis. Fit error bands correspond to variation at the 95% confidence interval. The solvent–accessible surface area (SASA) calculated with a 0.1 nm probe is also provided for each conformer. In this case, the bands correspond to plus/minus one standard deviation for the ensemble composed of the initial equilibrium conformations for the NEMD simulation.

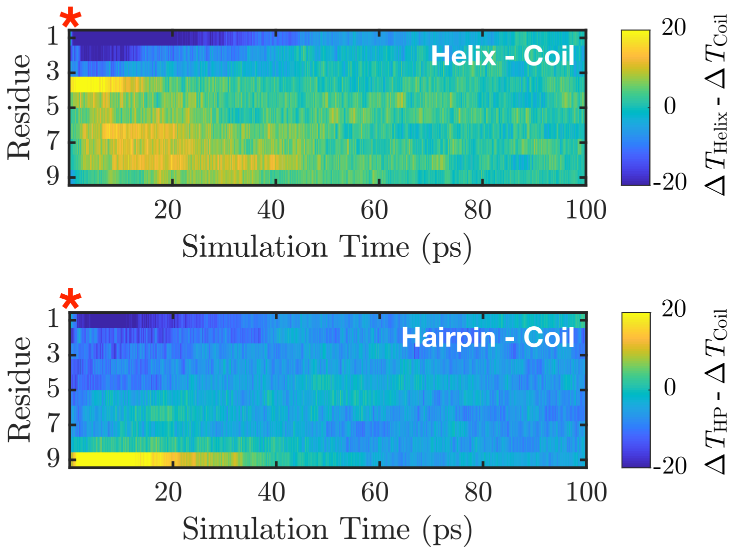

Supplementary Figure 17. Variation between transport profiles. Conformational dependence of the heat transport profile for helical, hairpin, and extended coil Aib_{10} ensembles during cooling simulations at $T_\text{B} = 230.0$ K. Raw data are quantified through the ensemble–averaged temperature elevation $\langle T_j \rangle$ over the bath temperature $\Delta T_{\text{B},j} = \langle T_j \rangle - T_\text{B}$ at residue j for designated conformational populations (following Fig. 1 of the main text). Plots correspond to a difference map of these elevations between helical and coiled ($\Delta T_{\text{Helix},j} - \Delta T_{\text{Coil},j}$), as well as hairpin and coiled ($\Delta T_{\text{HP},j} - \Delta T_{\text{Coil},j}$) structures. Heat redistribution throughout the peptide is more efficacious within helical and hairpin ensembles. For plotting purposes, the upper and lower temperature elevation (e.g., $\Delta T_{\text{B},j}$) bounds are a cutoff for all values lying outside the range. The heater site is denoted by a red asterisk.
